# Variations in the Root Form and Root Canal Morphology of Permanent Mandibular First Molars in a Sri Lankan Population

**DOI:** 10.1155/2015/803671

**Published:** 2015-08-13

**Authors:** Roshan Peiris, Uthpala Malwatte, Janak Abayakoon, Anuradha Wettasinghe

**Affiliations:** ^1^Department of Basic Sciences, Faculty of Dental Sciences, University of Peradeniya, 20400 Peradeniya, Sri Lanka; ^2^Faculty of Dental Sciences, University of Peradeniya, 20400 Peradeniya, Sri Lanka; ^3^Royal Oman Air Force, Masirah Air Base, 113 Muscat, Oman; ^4^Department of Restorative Dentistry, Faculty of Dental Sciences, University of Peradeniya, 20400 Peradeniya, Sri Lanka

## Abstract

The present study was conducted to determine the number of roots and morphology of the root canal system of permanent mandibular first molars (M1) in a Sri Lankan population. Sample of 529 M1 teeth was used. The number of roots was examined and the lengths of the mesial and distal roots were measured to the nearest 0.01 mm. Vacuum injection protocol was used to inject China ink into the root canal system, making it transparent. Root canal morphology was recorded using Vertucci's classification. Presence of furcation canals, position of lateral canals, intercanal communications, level of bifurcation, and convergence of the root canal system were recorded. M1 showed three roots in 4.1% of the sample. Commonest root canal morphology of the mesial root was type IV and the distal root was type I. The level of bifurcation of the root canals was commonly observed in the cervical one-third of the root while convergence was observed in the apical one-third in both roots. Prevalence of three rooted mandibular first molars is less than 5%. Mesial root showed the most variable canal morphology. Prevalence of furcation canals was 1.5% while that of middle mesial canals was 0.2%.

## 1. Introduction

Successful root canal treatment depends on adequate debridement and filling of the entire root canal system [1–5]. If the dental surgeon fails to recognize the presence of an additional root canal, adequately remove the pulp tissue, and disinfect and obturate the root canals properly, it may cause the failure of the entire treatment, altogether bringing frustration to the clinician as well as the patient. Therefore, it is important to be familiar with the variations in the root canal morphology because such knowledge can help in the location and negotiation of the canals as well as proper subsequent intervention.

The study of root and canal morphology has endodontic [4, 6] and anthropological significance [5, 7–12]. Moreover, the root canal morphology varies greatly among different populations and even in different individuals within the same population [7–9].

Root canal morphology has been classified in different ways by several investigators in the literature [6, 13, 14]. Weine et al. [14] classified it into four types depending on the pattern of division of the main root canal of a tooth along its course from the floor of the pulp chamber to the root apex. Meanwhile, Vertucci [6] categorized the root canal morphology in a more descriptive manner into eight types. This classification has been widely used by many investigators to classify the canal system of different teeth [1, 7–9, 15].

The permanent mandibular first molar (M1) is typically presented with two well-defined roots, a mesiodistally flattened mesial root and a mostly straight and more rounded distal root [2]. With regard to the number of roots, the most relevant variable is the presence of a third distolingual root [1, 2]. Carabelli first described this macrostructure in 1844 [1]. It was called “radix entomolaris” [1, 16]. This is a supernumerary root located distolingually in mandibular molars [16]. It is in general smaller than the distobuccal and mesial roots and can be separated from or partially fused with these roots [1]. This variant has a frequency of less than 5% in the white Caucasians, Africans, Eurasians, and Indians, whereas in Mongoloids, it has been observed in 5–40% of the cases [5].

Several methods have been used to study the root canal configuration of M1. They include plastic resin injection, endodontic access and radiographs with files into the root canals, retrospective evaluation of radiographs, clearing of samples with and without ink injection, sectioning and macroscopic or scanning electron microscopy evaluation, computed tomography (CT), spiral CT, micro CT, and cone beam CT [2].

Commonly, M1 has two canals in the mesial root: mesiobuccal and mesiolingual. Occasionally, a middle mesial canal can be found in the groove between the mesiolingual and mesiobuccal canals with the incidence ranging from 1% [6] to 13.3% [17]. Four root canals were also observed in the mesial root [18, 19]. Meanwhile, in the distal root, the majority has one canal which is considerably larger and more oval in cross section than mesial root canals and follows a straighter course as well [20]. Two canals were observed in 15–17% of the cases [6, 13, 21] and three canals have been observed in 1.7% of the distal roots [17, 21]. Therefore, the clinician must always look for such extra orifices after cleaning and shaping the main root canals. Apart from these main canals, furcation canals have been reported in 32% of the cases [6].

Unsuccessful root canal treatments usually take place as a result of missed root canals which are left to act as infection foci. They can be missed due to not knowing the exact number of the root canals as well as not being aware of the prevalence of unusual root canal configurations. Additional canals that might be rare, yet present in certain situations, may remain without intervention. Therefore, the main objectives of the present study were to find out the number of roots, morphology of the root canal system of the mesial and distal roots, and prevalence of the furcation and middle mesial canals of M1 in a Sri Lankan population. In addition, the exact position of the division or convergence of the root canals in relation to the length of the root was also investigated. This was considered as it could be useful in detecting extra canals divided from the main root canal. This research will help the dental practitioners in correctly diagnosing individual case with respect to the above mentioned attributes. The results obtained could be of value in order to execute the endodontic treatment in these teeth with greater confidence and accuracy with minimal hassle bringing much benefit to the patient at the end.

## 2. Materials and Method

A sample of 529 M1 teeth was collected from patients within the age range of 30–70 years. Teeth were extracted due to several reasons such as dental caries, periodontal disease, and prior undergoing of prosthodontic treatments, from various hospitals island wide. All the subjects enrolled in this research responded to an informed-consent protocol. The Faculty Research Committee of the Faculty of Dental Sciences, University of Peradeniya, Sri Lanka, approved this research and it conforms to the provisions of the Declaration of Helsinki in 1995 (as revised in Edinburgh 2000). The teeth, which were verified as M1 by crown morphology, were included in the study.

Teeth were washed immediately after extraction and stored in either water or normal saline for the purpose of prevention of desiccation. They were boiled in 5% NaOH for five minutes and then cleaned with 10% NaOCl to remove organic debris on the surface. Deposits such as calculus and bone fragments were removed by scaling and polishing. Each specimen was examined visually under a quartz-halogen light with the aid of a hand lens. The morphology of the root canals was recorded following Turner's classification [22]. The lengths of mesial and distal roots were measured using a digital vernier calliper to the nearest 0.01 mm.

After recording the external root morphology of the teeth, vacuum injection protocol described by Yoshiuchi et al. in 1972 [23] was used to inject the ink into the root canal system of each tooth and make the tooth transparent in order to visualize the canal morphology. Briefly described, access cavities were prepared in all teeth to expose the canal orifices to allow proper infiltration of the ink into the canal system. China ink was injected into the pulp cavity under high pressure two to three times using a vacuum injector. Teeth were then thoroughly cleaned with water to remove any ink on the surfaces and demineralized for five days in 5% nitric acid at room temperature (25°C). The nitric acid solution was changed every day. To test the reliability of the demineralization procedure, teeth were tested for softness by inserting a needle into the coronal region of the root. After demineralization, the teeth were rinsed in running water for 24 hours and then dehydrated using ascending concentrations of ethanol (70%, 80%, 90%, 95%, and 100%) for 5 days. Finally, they were rendered transparent by immersing them in a solution containing benzoic acid mixed with benzene and methylsalycylate in a ratio of 5 : 1 for 2-3 days. At the end of this procedure, all of the samples appeared transparent. The teeth, where the ink had not been infiltrated into the root canal system, were discarded.

**Figure 1:**
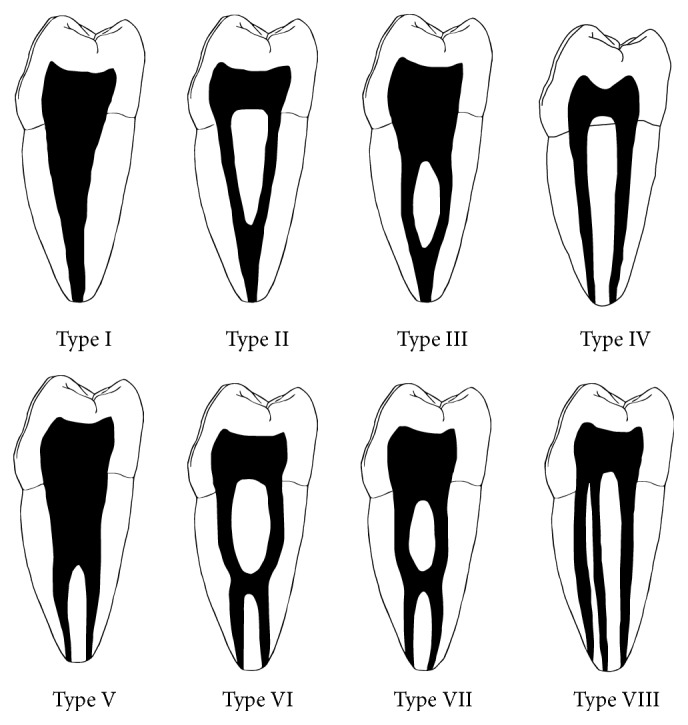
Vertucci's classification of root canal morphology [6].

The cleared specimens were examined under a dissecting microscope at ×10 magnification. The number and type of root canals were recorded using Vertucci's classification [6] of root canal morphology (Figure 1). The presence and the number and position of the lateral canals, intercanal communications, and furcation canals were also recorded. Mesial and distal roots were approximately divided into three equal segments along the length of the root as apical, middle, and cervical 1/3 s using a marker pen. When there was more than one root canal, in order to record the level of the bifurcation or convergence from the main root canal, the exact position was recorded with respect to these segments.

A test of consistency of the observer in assessing root canal types was done by reexamining the mesial root of 50 randomly selected molars and then comparing this test to the original canal assessment. Mesial root was selected because it showed the most variable and complicated canal morphology. The mean lengths of the mesial and distal roots were analyzed. The prevalence of number of roots with special emphasis on the radix entomolaris and the root canal types of the mesial and distal roots, as well as the evidence of the furcation canal, were calculated. SPSS (version 12, IBM corporation, New York, USA) software was used for the statistical analysis.

## 3. Results

In the present study, 95.8% of the M1 showed to have two roots while 4.1% showed to have three roots. The average lengths of the mesial and distal roots were 14.15 mm and 12.90 mm, respectively.

**Figure 2:**
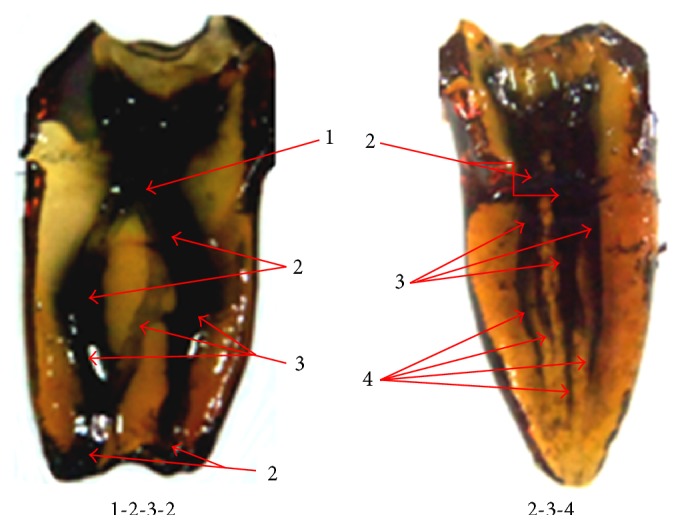
Additional canal configurations observed.

The majority of the mesial root showed Vertucci's type IV canal morphology (36.1%). In addition, type V canal configuration was observed in 32.9% and type II in 15% of the cases. Meanwhile, in the distal root, type I (65.0%) was the commonest and type V and type III were seen in 12.9% and 9.5% of the cases, respectively. Atypical canal configurations were more common in the mesial (3.6%) than distal root (1%) (Figure 2). In the third root, all the canals showed a type I canal morphology (Table 1).

The prevalence of lateral canals was most common at the apical third of both mesial and distal roots (99.3% and 100%, resp.) (Table 2). Intercanal communications were most prevalent at the apical third of the mesial roots (54%) and middle third of the distal root (51.5%) (Table 3).

When the canal morphology was Vertucci types II, III, V, VI, and VII, the level of bifurcation of the mesial and distal root canals were commonly observed at the cervical third, while the level of convergence was commonest at the apical third of the root (Tables 4 and 5).

Furcation canals were evident in 1.5% (*n* = 7) of the sample, while the middle mesial canals were found in 0.2% (*n* = 1) of the sample.

## 4. Discussion

According to Carlsen [24], the primary elements of the root complex are root cones and supernumerary root structures. For consistency with other publications [22, 25], we used the term “radical” rather than “cone” to refer to unseparated root like divisions. When a root has two or more radicals, the individual root elements may be divided either completely or incompletely. In completely separated roots, radicals are completely divided by interradicular processes at some point along the total length of a root and the result is two or more separated roots. When radicals are incompletely divided, owing to only the minimal penetration of the interradicular processes, superficial developmental grooves delimit the boundaries of the radicals. Therefore, it is possible that, in an incompletely separated root, although the root is not divided externally, the root canal system is divided internally.

In mandibular molars, two root components always occur, mesial and distal. These two root components are often separated by interradicular processes. In the first molar, each root component is composed of two radicals which are the primary elements of the root complex. Sometimes, mesial root component may have three radicals, namely, buccal, middle mesial, and lingual. Root radicals were usually nonseparated throughout the cervicoapical extension. However, as mentioned by Carlsen in 1987, in M1 and occasionally in the second molar, the two root radicals may be separated apically [25]. Scott and Turner [25] further noted that when radicals were completely divided by root bifurcation at some point along the root length of root, the result was two or more separate roots. However, in M1, complete division of radicals was not common. In the present study, a middle mesial canal was seen in a mesial root M1 tooth. This can be explained as this mesial root was composed of three root radicals and the individual radicals have been incompletely divided. Therefore, the mesial root was not separated externally but the root canal was separated into three canals internally representing the three root elements.

Furcation canals are formed during tooth development as a result of entrapment of periodontal vessels during the fusion of the diaphragm which becomes the floor of the pulp chamber [26]. These canals may cause primary endodontic lesions in the furcation of multirooted teeth, which provide a rationale for placing an adhesive restoration on the floor of the pulp chamber to prevent furcal breakdown [16]. Although the prevalence of furcation (1.5%) and middle mesial canals (0.2%) in this study was much lower compared to the study done by Vertucci in 1984 [6] (32% and 1%, resp.), clinicians should always look for the possibility. It was reported that removal of the obturating material at the furcation region and proper sealing of the area with an adhesive restorative material could reduce the incidences of infection of the treated tooth through this route [27].

Radix entomolaris was observed in 4.1% of the cases while 95.8% of cases showed only the two roots, mesial and distal. Meanwhile, another study done in Sri Lanka by Fonseka et al. [28] reported two and three rooted M1 in 97% and 7% of the teeth, respectively. A similar study done by Peiris [9] revealed two rooted molars in 94.4% of the cases and three rooted molars in 5.6% of another Sri Lankan population. A study done on Hindus in Singapore had revealed 0.2% of the cases of three rooted mandibular first molars [29]. Lukacs [30] recorded an occurrence of 5.6% of M1 with three roots in a neolithic population in Pakistan [25]. In addition, Gu et al. [31] and Haung et al. [32] found an extra distolingual root in mandibular first molars in a Chinese and a Taiwanese population as 23% and 25.3%, respectively. In a similar study, Song et al. [33] showed a prevalence of 24.5% of third distolingual root in a Korean population. Interestingly, Rwenyonyi et al. [34] did not find any case of M1 with three roots in 224 cases studied of an Ugandan population. Therefore, the occurrence of radix entomolaris in M1 in the present study agrees with the result of other Sri Lankan and South Asian studies. However, it was far different from those of Sino American populations such as Chinese, Japanese, Korean, and Taiwanese, which showed a prevalence of more than 5%. Furthermore, the radix entomolaris was considered as a supernumerary structure and therefore it cannot be explained by the normal root developmental pattern of M1.

On the other hand, with regard to root canal morphology of M1, Vertucci [6] reported the prevalence of type I canal configuration in the distal root to be 70.0%, with 85.0% having one foramen at the apex in an American white population. Moreover, type IV canal form was seen in 51.0% of the mesial roots, type II in 28.0%, and type I in 12.0% with 40.0% and 59.0%, having one and two apical foramina, respectively. Sert and Bayirli [4] encountered type I canal configurations in 53.5% and type II and type III in 12.5% and 21.0%, respectively, in the distal roots with 87.0% having one apical foramen in a Turkish population. They also revealed that type IV form was found in 43.0% and type II in 44.0% in the mesial root of M1 having one apical foramen found in 51.0% and two in 45.0% of the cases. Furthermore, Peiris [9] recorded types IV and II canal configuration in 57.3% and 26.5% of the mesial roots, respectively, with one apical foramen in 33.3% and two foramina in 61.6% of the cases. Type I canal configuration was found in 73.2% of the distal roots with one apical foramen in 80.3% of the cases. In the present sample, 65.0% of distal root of M1 had type I canal morphology and 79.2% were presented with one foramen at the apex. In addition, the prevalence of type IV and type V canal configuration in the mesial root was 36.1% and 32.9%, respectively, whereas one apical foramen was observed in 25.9% and two foramina were observed at the apex in 69.4% of the cases. Thus, the result of the present study of Sri Lankans, especially the number of apical foramina in the distal root of M1, compares favorably with other studies done on Sri Lankan, American white, and Turkish populations. Meanwhile, Gulabivala et al. [5] who investigated canal configurations of M1 in a Thai population reported that 24.5% of distal root had type IV canal form and 27.1% showed two foramina at the apex. Gulabivala et al. [35] who performed a similar study on Burmese population determined the incidence of type IV canal configuration in 18.7% and prevalence of two apical foramina to be 25.9%. Furthermore, Walker [10] found the incidence of two apical foramina in the distal root of M1 to be 28.0% in a southern Chinese population. Therefore, the frequency of number of apical foramina in the distal root of M1 of Thai, Burmese, and Chinese is very different from those of Sri Lankan population. These findings seem to be related to the frequent occurrence of three rooted M1 in Thai and Burmese, who reflect cultural mix of Chinese and Indian origins [5, 35], and Chinese populations [10].

Meanwhile, most lateral canals were evident at the apical 1/3 of both roots in the present study (mesial root: 99.3% and distal root: 100%). Intercanal communications were most prevalent at the apical 1/3 of the mesial root (54%) and middle 1/3 of the distal (51.5%). The study done by Vertucci [6] reported 54.4% and 57.9% of the lateral canals at the apical 1/3 of mesial and distal roots, respectively. In the same study, intercanal communications were most prevalent at the middle 1/3 of both roots having 75% in the mesial root and 72% in the distal. Peiris et al. [8] reported presence of lateral canals at the apical 1/3 in 90.9% and 83.3% of mesial and distal roots, respectively. Furthermore, intercanal communications were recorded at the middle 1/3 of the root in 80.6% and 58.3% of mesial and distal roots, respectively. It was found by several studies that these canals pave the way for the infectious microorganisms to enter into the root canal system even if the main canals were three dimensionally well obturated [27, 36]. Although these minute details of the root canal system cannot be obturated using gutta-percha, they can be made patent using various root canal irrigants such as 5% sodium hypochloride and EDTA (ethylene diamine tetra-acetic acid) and thus allowed to be filled by the root canal cement material. This blocks the pathways of reinfection of the root canal system via periodontal ligament space [16]. Knowing the above data will encourage the clinicians to use standardized root canal irrigating systems and technology like ultrasonic irrigation to agitate irrigating medium within the root canal system to enhance its cleansing.

The level of bifurcation of most of the mesial and distal root canals was at the cervical 1/3 (97% and 81.8%, resp.). Therefore, it is about 4.71 mm and 4.30 mm from the cervical margins of the mesial and distal roots, respectively. The level of convergence was at the apical 1/3 in the majority of the canals in both roots (82.6% in mesial and 92.8% in distal roots). Thus, it is about 9.43 mm and 8.70 mm from the cervical margins of the mesial and distal roots, respectively. Although there were no studies in the literature to compare these results, we anticipate that the level of bifurcation and convergence of root canals in the mesial and distal roots of the mandibular first molar may vary in different populations. Therefore, it is important to investigate these aspects in other populations.

In the meantime, it is important to mention that the clearing technique used in the present study has several drawbacks over more modern micro-CT and cone beam CT techniques to study root canal morphology. Clearing technique is a destructive method and occasionally very narrow canals may not be stained properly with this technique. In addition, it is time-consuming and extracted teeth are required to use the technique.

## 5. Conclusions

The prevalence of three rooted mandibular first molars is less than 4.1% in the present Sri Lankan sample. Mesial root shows the most variable canal morphology with a prevalence of 1.5% furcation canals and 0.2% middle mesial canals. In the mesial root, root canals are commonly bifurcated and converged at the cervical and apical 1/3, respectively. This knowledge would be of great benefit to the clinician to look for the possibility of the bifurcation in what seems like a single straight root canal and to have an idea of the converging point. These will avoid any unwanted procedural errors during canal preparations like canal transportation, zipping, and even perforations.

## Figures and Tables

**Table 1:** Root canal morphology of the specimens.

	I	II	III	IV	V	VI	VII	VIII	A
Mesial root (*n* = 385)	4 (1.03%)	57 (14.81%)	38 (9.87%)	140 (36.36%)	127 (32.99%)	1 (0.26%)	1 (0.26%)	0	14 (3.64%)
Distal root (*n* = 386)	252 (65.28%)	18 (4.66%)	37 (9.59%)	22 (5.70%)	50 (12.95%)	1 (0.26%)	0	0	4 (1.04%)

A: additional canals configurations.

**Table 2:** Prevalence of lateral canals (percentage from the total number of roots).

	Cervical 1/3	Middle 1/3	Apical 1/3
Mesial root	0	1 (0.26%)	157 (40.78%)
Distal root	0	0	148 (38.34%)

**Table 3:** Prevalence of intercanal communications (percentage from the total number of roots).

	Cervical 1/3	Middle 1/3	Apical 1/3
Mesial root	10 (2.60%)	53 (13.77%)	74 (19.22%)
Distal root	0	17 (4.40%)	16 (4.15%)

**Table 4:** Level of bifurcation of root canal.

Vertucci's root canal type	Mesial root			Distal root		
	Cervical 1/3	Middle 1/3	Apical 1/3	Cervical 1/3	Middle 1/3	Apical 1/3
III	37 (97.4%)	1 (2.60%)	0	34 (91.90%)	3 (8.1%)	0
V	125 (98.4%)	2 (1.6%)	0	38 (76.0%)	10 (20.0%)	2 (4.0%)
VI	0	0	1 (100%)	0	1 (100%)	0
VIII	0	0	1 (50%)	—	—	—

**Table 5:** Level of convergence of the root canal.

Vertucci's root canal type	Mesial root			Distal root		
	Cervical 1/3	Middle 1/3	Apical 1/3	Cervical 1/3	Middle 1/3	Apical 1/3
II	0	12 (20.7%)	46 (79.3%)	0	1 (5.6%)	17 (94.4%)
III	0	3 (7.9%)	35 (92.1%)	0	2 (5.4%)	35 (94.6%)
VI	0	1 (100%)	0	0	1 (100%)	0
VII	0	1 (100%)	0	—	—	—
